# Evaluation of the Effects of Mouthwash on the Morphology and Cell Viability of Osteoblast-Like Cells

**DOI:** 10.1155/2022/5884974

**Published:** 2022-02-04

**Authors:** JaeHyung Lim, Ji Eun Lee, Chang-Joo Park, Jun-Beom Park

**Affiliations:** ^1^Division of Oral and Maxillofacial Surgery, Department of Dentistry, Korea University Ansan Hospital, Ansan 15355, Republic of Korea; ^2^Department of Periodontics, College of Medicine, The Catholic University of Korea, Seoul 06591, Republic of Korea; ^3^Division of Oral & Maxillofacial Surgery, Department of Dentistry, Hanyang University College of Medicine, Seoul 04763, Republic of Korea

## Abstract

This study evaluated the effects of multiple mouthwashes on the cellular viability or the morphology of preosteoblasts. Mouse calvarial osteoblast-like cells were cultured and treated with mouthwashes of (1) benzydamine hydrochloride; (2) cetylpyridinium chloride and benzalkonium chloride; (3) methyl salicylate, menthol, eucalyptol, and thymol; and (4) sodium fluoride, xylitol, and chitosan. The treatment times were 30 seconds, 90 seconds, and 270 seconds. Cell morphology was evaluated with a microscope, and the viability of the treated cells was analyzed quantitatively using a commercially available kit. The untreated control group exhibited well-stretched fibroblast-like morphology. Treatment with mouthwash resulted in morphological changes in all groups. Treatment with sodium fluoride resulted in more noticeable changes. Treatment with mouthwash for 30 seconds produced a significant decrease in cell viability. An increase in time to 90 and 270 seconds did not produce additional noticeable changes. To conclude, commercially available mouthwashes created changes in cell morphology and decreased the cell viability of osteoblast-like cells irrespective of ingredients and treatment time.

## 1. Introduction

Mouthwash is used frequently in daily life and has the advantage of reaching areas that are not easily accessible with a toothbrush [1, 2]. The use of mouthwash can aid people with daily oral hygiene [3]. Mouthwash has been used to heal soft tissue, reduce gingivitis, control plaque, reduce dental caries, control bad breath, and whiten teeth [4, 5]. Moreover, chemical plaque control is the most commonly recommended means of oral hygiene after periodontal surgery [6]. A variety of mouthwashes is available by prescription or over the counter [7].

There are several main active components for mouthwashes including cetylpyridinium chloride, sodium fluoride, and essential oils [8]. Benzydamine has been recommended for prophylaxis of oral mucositis in head and neck cancers [9]. Cetylpyridinium chloride is a quaternary ammonium compound and has been used to reduce dental plaque and gingivitis [10]. Sodium fluoride has been reported to have an anticarious effect, and the use of mouthwash containing sodium fluoride can enhance remineralization of teeth [11]. Mouthwash containing methyl salicylate, menthol, eucalyptol, and thymol has been used as an adjunct to daily oral hygiene care due to the antiplaque and antigingivitis effects [12]. Previous reports have studied the possibilities of cytotoxic effects of mouthwash on cells [6, 13–15]. This study examined the effects of multiple mouthwashes on the viability or morphology of osteoblast-like cells.

## 2. Materials and Methods

### 2.1. Cell Culture

Mouse calvarial osteoblast-like cells (MC3T3-E1) were deposited in 96-well plates at a density of 6.25 × 10^3^ cells/well and maintained in *α*-minimum essential medium (*α*MEM, Welgene, Daegu, Korea) supplemented with 10% fetal bovine serum (Thermo Scientific, Logan, UT, USA), penicillin 100 U/mL, and streptomycin 100 *μ*g/mL (Gibco, Invitrogen, Carlsbad, CA, USA). The cultures were kept in a humidified atmosphere with 5% CO_2_ and 95% air at 37°C.

### 2.2. Evaluation of Cell Morphology after Use of Mouthwash

**Figure 1:**
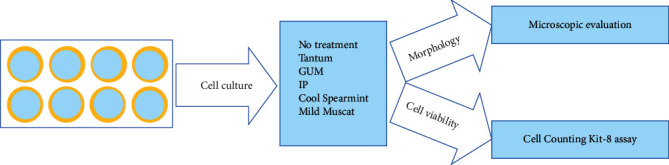
Diagram showing the overview of the study design.

Figure 1 shows the overview of the study design. Six mouthwashes were tested in this study: (1) benzydamine hydrochloride (150 mg/100 mL; Tantum, Sama Pharm Co. Ltd., Wonju-si, Gangwon-do, Korea); (2) cetylpyridinium chloride (50 mg/100 mL) and benzalkonium chloride **(**GUM, Sunstar Inc., Osaka, Japan); (3) methyl salicylate, L-menthol, eucalyptol, and thymol (IP, 3M, St. Paul, MN, USA); (4) sodium fluoride (0.2 mg/1 mL**)**, xylitol, and chitosan (Cool Spearmint, 3M); and (5) sodium fluoride, xylitol, and chitosan (Mild Muscat, 3M). The treatment times were 30 seconds, 90 seconds, and 270 seconds. An untreated culture sample served as the control. The morphological changes were observed under an inverted microscope (Leica DM IRM, Leica Microsystems, Wetzlar, Germany) after each treatment.

### 2.3. Quantitative Determination of Cell Viability

The cell viability of the osteoblast-like cells was analyzed quantitatively by a Cell Counting Kit-8 (Dojindo Molecular Technologies Inc., Rockville, MD). A water-soluble tetrazolium salt-8 solution was added to the culture and incubated for four hours. The amount of generated formazan was analyzed as absorbance at a 450 nm wavelength using a microplate spectrophotometer system (BioTek, Winooski, VT).

### 2.4. Statistical Analysis

The results were presented as the mean ± standard error of the mean of the experiments. A test of normality and the equality of variances in the samples were conducted. Two-way analysis of variance was used for evaluation of the effects of application time and types of gargles using a commercially available program (SPSS 12 for Windows, SPSS Inc., Chicago, IL, USA) with a level of significance at 0.05.

## 3. Results

### 3.1. Evaluation of Cell Morphology and Cell Viability

**Figure 2:**
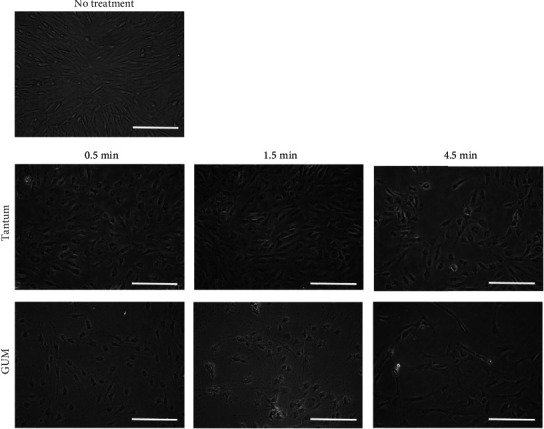
Evaluation of cellular morphology after treatment with benzydamine hydrochloride and cetylpyridinium chloride. The scale bar indicates 200 *μ*m.

**Figure 3:**
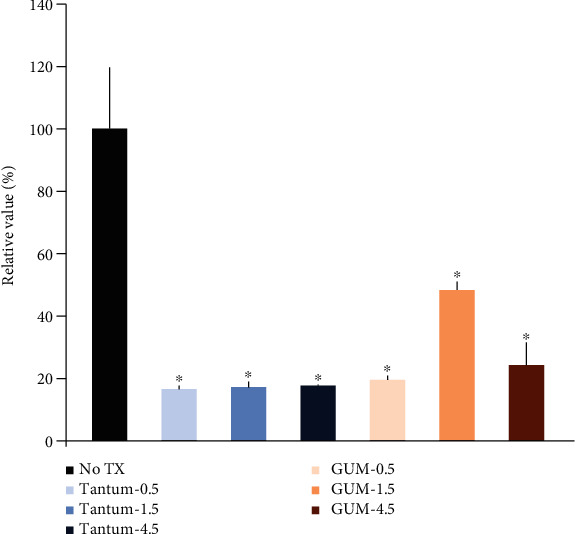
Cellular viability using Cell Counting Kit-8 after treatment with benzydamine hydrochloride and cetylpyridinium chloride.

In the microscopic evaluations, the untreated cells attached to the culture plate exhibited well-organized fibroblast-like actin cytoskeletons. Treatment of the osteoblast-like cells with Tantum resulted in an alteration in morphology (Figure 2). Treatment for longer times resulted in a more rounded shape. Similar trends were achieved in the GUM group. Alterations in cytoskeletal organization and progressive detachment from the culture plate were observed with longer treatment time (Figure 2). The relative cell viability was 16.6% ± 1.2%, 17.1% ± 1.8%, and 17.6% ± 0.4% for Tantum at 30, 90, and 270 seconds, respectively, when the untreated control was considered 100% (100.0% ± 19.5%) (Figure 3). The mean cell viability for the GUM group was 19.8% ± 1.2%, 48.2% ± 2.9%, and 24.3% ± 7.2% at 30, 90, and 270 seconds, respectively.

**Figure 4:**
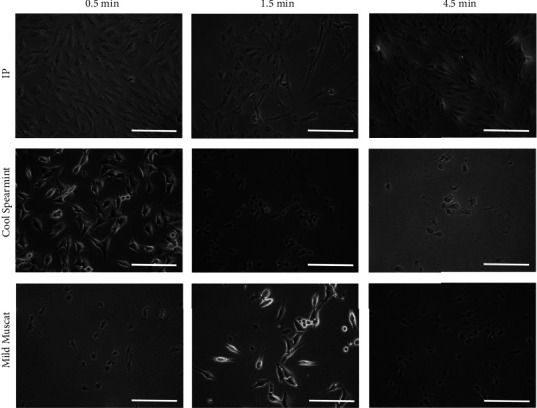
Evaluation of cellular morphology after treatment. The scale bar indicates 200 *μ*m.

Cellular morphology after treatment with 3M mouthwashes is provided in Figure 4. Alterations in cytoskeletal organization were seen irrespective of the formulations. Agglomeration and detachment of the cells from the culture plate were noted.

**Figure 5:**
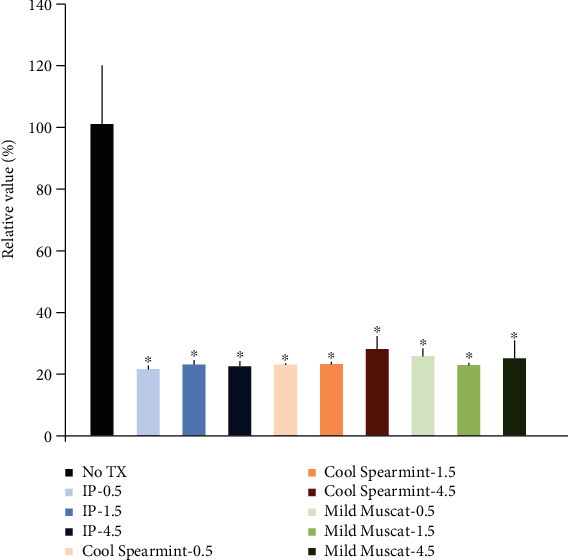
Cellular viability using the Cell Counting Kit-8 after treatment with mouthwashes containing methyl salicylate, L-menthol, eucalyptol, and thymol or sodium fluoride, xylitol, and chitosan.

The relative cell viability for IP was 16.9% ± 0.8%, 18.6% ± 1.5%, and 18.2% ± 1.6% at 30, 90, and 270 seconds, respectively, when the untreated control was considered 100% (Figure 5). The mean cell viability for the Cool Spearmint group was 18.7% ± 0.3%, 18.8% ± 0.7%, and 23.8% ± 4.3% at 30, 90, and 270 seconds, respectively. The cell viability for the Mild Muscat group was 21.5% ± 2.5%, 18.4% ± 0.7%, and 20.7% ± 6.0% at 30, 90, and 270 seconds, respectively.

## 4. Discussion

This study showed that treatment with mouthwashes resulted in morphological changes and reduction in cell viability in all groups.

The cytotoxic effects of mouthwashes have been previously reported [6, 13]. A previous in vitro study showed that undiluted mouthwashes induced near-complete cell death of human gingival and periodontal ligament fibroblasts 24 hours after only a 60-second treatment [6]. Dilutions of 15% to 20% for both essential oil mouthwashes resulted in reduction of cell death to 50%, and dilutions of 10% to 15% of essential oils did not reduce cell migration [6]. A previous report tested various antiseptic agents on in vitro human gingival fibroblast proliferation [13]. The remaining viable cell density after application of 0.2% chlorhexidine was 35.2%, and 0.15% benzydamine hydrochloride exhibited weaker cytotoxic effects, with the lowest cytotoxic effect in the essential oil group [13]. This present study showed that treatment with mouthwash for 30 seconds resulted in 20% to 30% cell viability.

Chemical plaque control is the most commonly recommended means of oral hygiene after periodontal surgery [6]. However, mouthwashes should not be considered a substitute for daily brushing and flossing [16, 17]. Swallowing or ingesting mouthwash should be avoided whenever possible [18] and can cause vomiting, nausea, or intoxication [19]. Children, especially young children, should not use mouthwash unless required or prescribed by a dental professional [20]. Manufacturers recommend specific durations or sequences of use depending on concentration and ingredients [21]. It is a matter of personal preference whether to rinse before or after brushing [22]. Toothpaste ingredients such as calcium hydroxide or aluminum hydroxide can form complexes with fluoride ions, reducing the effectiveness of mouthwashes [23, 24]. If these ingredients are present in the toothpaste, it is recommended to rinse vigorously with water before using the mouthwash [25]. The use of diluted mouthwash can be considered because dilution of mouthwash including essential oils retained most of the antibacterial effects with minimal detrimental effects on human gingival and periodontal ligament fibroblasts [6].

Increasing evidence suggests that acetaldehyde, the first and genotoxic metabolite of ethanol, mediates the carcinogenicity of alcoholic beverages [26]. Ethanol is contained in a number of ready-to-use mouthwashes, typically between 5% and 27% volume [26]. The doses and administration times of antiseptics should be controlled carefully during dental application [13]. Further studies are required to determine the optimal application time and concentration of this antimicrobial agent to maximize reduction of the bacterial load and minimize cytotoxicity to the surrounding cells [15, 27].

According to previous publications on implants, the maintenance/management of implants is becoming more important than topics related to implant placement or osseointegration [28]. Among these subjects, especially in relation to peri-implantitis, it is essential to reduce bacteria and inflammation while delaying and preventing bone resorption/destruction [29]. In relation to the treatment of medication-related osteonecrosis of the jaw, which has been a major issue in dentistry for over 10 years, oral antimicrobial rinse cannot be left out [30, 31]. Even now, it is a reality that systemic antibiotics, mechanical cleaning and/or removal, and local chemical rinsing are recommended for treatment of these bone-related lesions [32]. With regard to chemical rinse, in order to preserve vital bone as much as possible and reduce infection/inflammation, it is necessary to study the correct concentration and application time to reduce bacteria/inflammation while properly maintaining and restoring osteoblast activity. In relation to peri-implantitis, it is essential to reduce bacteria and inflammation while delaying and preventing bone resorption/destruction [33, 34]. In addition, in relation to the treatment of medication-related osteonecrosis of jaw, oral antimicrobial rinse cannot be omitted [35]. In the future, similar experiments or in vivo experiments can be conducted to compare with these results and this result may serve as a baseline.

## 5. Conclusions

This study examined the effects of mouthwashes on the viability or morphology of osteoblast-like cells. The results showed that treatment with mouthwashes resulted in morphological changes and reduction in cell viability in all groups, with more noticeable changes by sodium fluoride. Collectively, commercially available mouthwashes resulted in changes in cell morphology and decreased cell viability of osteoblast-like cells irrespective of ingredients and treatment time.

## Data Availability

All data generated or analyzed during this study are included in the published article.
